# Inhibition of the NKCC1/NF-κB Signaling Pathway Decreases Inflammation and Improves Brain Edema and Nerve Cell Apoptosis in an SBI Rat Model

**DOI:** 10.3389/fnmol.2021.641993

**Published:** 2021-03-31

**Authors:** Yating Gong, Muyao Wu, Jinchao Shen, Jiafeng Tang, Jie Li, Jianguo Xu, Baoqi Dang, Gang Chen

**Affiliations:** ^1^Department of Rehabilitation, Zhangjiagang TCM Hospital Affiliated to Nanjing University of Chinese Medicine, Suzhou, China; ^2^Department of Anesthesiology, Zhangjiagang TCM Hospital Affiliated to Nanjing University of Chinese Medicine, Suzhou, China; ^3^Department of Intensive Care Unit, Zhangjiagang TCM Hospital Affiliated to Nanjing University of Chinese Medicine, Suzhou, China; ^4^Department of Neurosurgery, The First Affiliated Hospital of Soochow University, Suzhou, China; ^5^Department of Neurosurgery and Brain and Nerve Research Laboratory, The First Affiliated Hospital of Soochow University, Suzhou, China

**Keywords:** surgical brain injury, NKCC1, NF-κB, neuroinflammation, brain edema, apoptosis

## Abstract

Surgical brain injury (SBI) triggers microglia to release numerous inflammatory factors, leading to brain edema and neurological dysfunction. Reducing neuroinflammation and protecting the blood-brain barrier (BBB) are key factors to improve the neurological function and prognosis after SBI. Na^+^-K^+^-Cl^–^ cotransporter 1 (NKCC1) and nuclear factor κB (NF-κB) have been implicated in the secretion of inflammatory cytokines by microglia in brain injury. This study aimed to establish the role of NKCC1 in inducing inflammation in SBI, as well as to determine whether NKCC1 controls the release of interleukin-1β (IL-1β), interleukin-6 (IL-6), and tumor necrosis factor-α (TNF-α) via phosphorylation of NF-κB in microglia, thus affecting BBB permeability and neuronal cell apoptosis. Male Sprague-Dawley (SD) rats were used to establish an SBI model. This study revealed that compared with the sham group, the expression levels of p-NKCC1, p-p65-NF-κB, and related inflammatory factor proteins in SBI model group significantly increased. After p-NKCC1 was inhibited, p-p65-NF-κB, IL-6, IL-1β, and TNF-α were downregulated, and nerve cell apoptosis and BBB permeability were significantly reduced. These findings suggest that the SBI-induced increase in p-NKCC1 exacerbates neuroinflammation, brain edema, and nerve function injury, which may be mediated by regulating the activity of p65-NF-κB that in turn influences the release of inflammatory factors.

## Introduction

Brain surgery plays a major role in studying the etiology and treatment of brain diseases (Hamard et al., 2016). At the same time, due to incision, electrocoagulation, bleeding, and other invasive operations in the process of brain surgery, there will be some damage to the brain tissue around the operation site, which is called surgical brain injury (SBI) (Akyol et al., 2018). Studies have shown that nearly one-fifth of patients experience serious complications after craniocerebral operation (Sherchan et al., 2016). After brain injury caused by various reasons, immune cells are activated, triggering the release of pro-inflammatory cytokines, thus resulting in the formation of an inflammatory environment in the brain, which is a feature of many brain pathologies. When inflammatory signals are activated, multiple cell signals in the brain change, which may eventually lead to neurological dysfunction and cell apoptosis (Lima Giacobbo et al., 2019). Neuroinflammation is a major adverse reaction that occurs after brain injury that may lead to brain edema, nerve cell apoptosis, as well as aggravated nerve function injury (Xiao et al., 2018). Microglia cells pertain to immune cells in the central nervous system. Generally, microglia are at rest. In brain injury, microglia are stimulated to activate the nuclear factor κB (NF-κB) signaling pathway, which induces cells to contribute to the generation of an inflammatory response and triggers microglia to secrete a various inflammatory factors, including interleukin-1β (IL-1β), interleukin-6 (IL-6), as well as tumor necrosis factor alpha (TNF-α), which then cause cellular neurotoxicity and lead to secondary brain injury (Wu et al., 2017b; Zhang et al., 2020a).

Previous studies have shown that inflammation can worsen brain edema (Adukauskiene et al., 2007). After brain injury, inflammatory cytokines such as IL-1β, IL-6, TNF-α, as well as oxidative stress mediators are upregulated, which then may result in apoptosis of large neurons and incur blood-brain barrier (BBB) damage, thereby exacerbating brain edema and nerve cell damage (Chen et al., 2014; Zhang et al., 2020b). Inflammation can increase cell permeability, disrupt the BBB, and lead to vasogenic brain edema; it also causes cell degeneration and apoptosis, and if necrotic and apoptotic cells are not immediately consumed by phagocytes, then these could release toxic signals to surrounding cells, leading to cytotoxic brain edema (Sherchan et al., 2017; Xiao et al., 2018).

Na^+^-K^+^-Cl^–^ cotransporter 1 (NKCC1) is expressed in glial cells, neurons, endothelial cells, and choroid plexus epithelial cells (Simard et al., 2010). The primary function of NKCC1 is to regulate the entry of Na^+^, K^+^, Cl^–^, and water into cells to regulate the transfer and invasion of inflammatory mediators and other related factors, which can lead to brain edema, inflammation, and secondary brain damage. NKCC1 is closely related to cerebral hemorrhage (Wu et al., 2020a), stroke (Wu et al., 2020b), epilepsy (Gharaylou et al., 2019; Zhang et al., 2020b), traumatic brain injury (Sudhakar et al., 2019), and other brain diseases. However, the activity of NKCC1 is mainly regulated by the STE20-related proline/alanine kinase (SPAK)/oxidative stress response 1 (OSR1) signaling pathway. After injury, SPAK/OSR1 is activated to phosphorylate downstream NKCC1 (Yan and Merlin, 2008). SPAK/OSR1, which is the upstream kinase of NKCC1, binds to NKCC1 through its conserved C-terminal domain, phosphorylates Thr203, Thr207, and Thr212 residues on NKCC1, thus promoting the increase of inflammatory cells and aggravating the inflammatory response after injury (Reid et al., 2013; Hung et al., 2020). Inhibition of NKCC1 downregulates the expression of intracellular NF-κB phosphorylation (Zhang et al., 2017), thus reducing the activation of inflammatory cells and cell volume-related functions and decreasing brain edema and nerve cell injury after brain injury (Hung et al., 2018; Gomez et al., 2019). Pro-inflammatory cytokines IL-1β, IL-6, and TNF-α also play an important role in the inflammatory response via this signaling pathway.

Neuroinflammation induced by SBI surgery can aggravate brain edema and postoperative nerve function injury (Huang et al., 2020a). Currently, the treatment of secondary brain injury after SBI is limited. Therefore, the development of endogenous therapeutic measures will be a safe way to reduce postoperative complications of SBI. Bumetanide (BUM) is a loop diuretic that acts by inhibiting NKCC1 and NKCC2. NKCC1 is expressed in the central nervous system and systemic organs, and BUM regulates the transmembrane Cl^–^ gradient of neurons by blocking NKCC1 (Kharod et al., 2019). BUM was proposed to provide an interesting therapeutic option for such brain diseases by reducing intraneuronal Cl^–^ levels and restoring GABAergic inhibition (Hampel et al., 2021). Currently, BUM has been proposed to inhibit NKCC1 to reduce intracranial Cl^–^ level, providing a treatment option for this kind of brain disease. BUM was identified and recognized nearly 40 years ago and has been used to treat hypertension and brain edema, with side effects limited to diuretics, hypokalemia and dehydration (Ben-Ari, 2017). The aim of this study was to investigate the relationship between NKCC1 and microglia cell-induced release of inflammatory factors after SBI, as the inhibition of NKCC1 can reduce p-p65-NF-κB activation, decrease the release of inflammatory factors, thereby reducing brain edema and nerve cell apoptosis as well as improving nerve function, indicating that it plays a role in brain protection.

## Materials and Methods

### Experiment Design and Grouping

**FIGURE 1:**
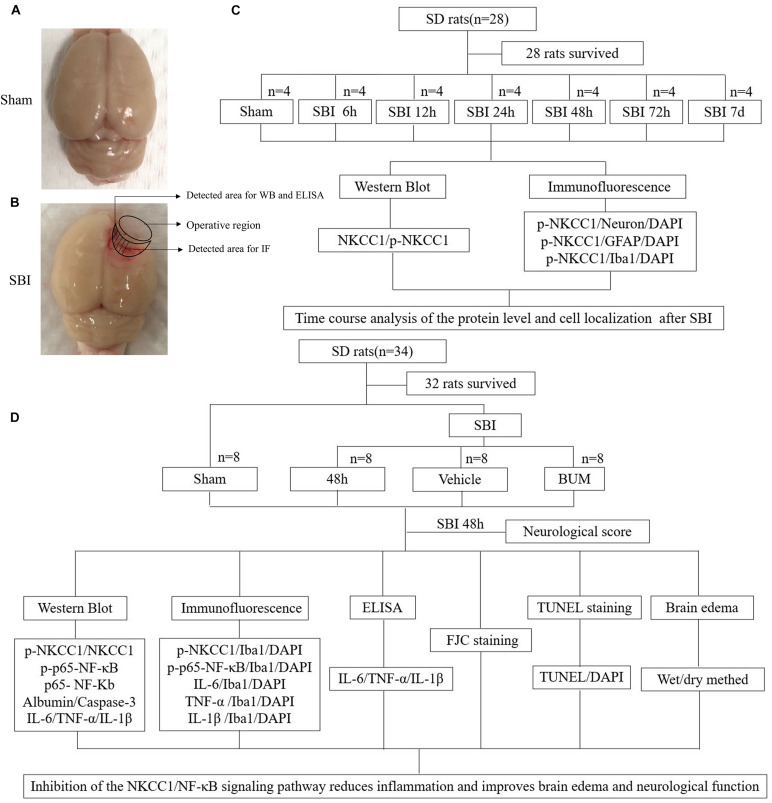
SBI model and experimental design. Brain tissues obtained from the perioperative area of the SBI group and from the same site in the sham group were assessed, some tissues were used for WB, IF staining, and ELISA **(A,B)**. NKCC1 and p-NKCC1 expression levels and locations of p-NKCC1 in nerve cells post SBI and determination of the optimal time point for the subsequent experiment **(C)**. Assessment of the effects of the NKCC1/NF-κB pathway post SBI and elucidation of potential mechanisms **(D)**.

Experiment 1: No significant difference in weight, exercise ability, and food intake were observed among groups. Time course analysis of NKCC1 and p-NKCC1 protein levels in 28 rats after SBI. A total of 28 rats (all survived) were randomly assigned to one of seven groups (*n* = 4 per group), namely, the sham operation group, and six experimental groups that were arranged in chronological order of 6 h, 12 h, 24 h, 48 h, 72 h, and 7 days post SBI operation. The rats were sacrificed at a time between 6 h and 7 days, which was 36 h after sham surgery. The brain tissue around the damaged area in each rat was collected. Western blotting (WB) of a portion of the brain tissues was conducted to determine the expression of NKCC1 and p-NKCC1, and the rest of the tissue samples was used for double immunofluorescence (IF) to assess the expression of p-NKCC1 (Figure 1C).

Experiment 2: To determine the role of p-NKCC1 in SBI, 32 rats (a total of 34 rats were used, of which 32 survived) were randomly assigned to one of the four groups, namely, sham, SBI, SBI + vehicle, and SBI + BUM. Based on the results of experiment 1, the rats were sacrificed 48 h post SBI, and brain tissues surrounding the damaged area were collected. Neurological examination of all groups was performed prior to death. Sixteen rats (four rats per group) were used in WB, IF, and enzyme-linked immunosorbent assay (ELISA). Brain tissues near the pre-lesion area were used in WB to determine p-NKCC1, p-p65-NF-κB, p65-NF-κB, albumin, caspase-3, IL-1β, IL-6, and TNF-α expression and in ELISA for IL-1β, IL-6, TNF-α expression. Tissues in the post-lesion area were processed for paraffin sectioning for TdT-mediated dUTP nick-end labeling (TUNEL) staining as well as Fluoro-Jade C (FJC) to assess nerve cell apoptosis and necrosis. Sixteen rats (four in each group) were evaluated for brain edema. The experiment was conducted with blinded experimenters (Figure 1D).

### Experimental Animals

All experiments received approval from the Institute of Animal Care Committee of Zhangjiagang Traditional Chinese Medicine Hospital (Zhangjiagang, China) and were conducted following the guidelines on the care and use of animals of the National Institutes of Health. We purchased male Sprague-Dawley (SD) rats (age: 8 weeks; weight: 320–350 g) from the Zhaoyan (Suzhou) New Drug Research Center. The rats were maintained under constant temperature and relative humidity, as well as were fed using a regular light/dark cycle. Food and water were provided *ad libitum*.

### Establishment of Experimental Rat SBI Model

A rat SBI model was used as earlier reported (Huang et al., 2020a). SD rats were anesthetized via intraperitoneal injection of sodium pentobarbital (40 mg/kg). The rats were then placed in a prone position and fixed onto a stereotaxic apparatus (Yuyan, China). An incision was made along the midline of the skin above the brain to expose the skull to identify the bregma. Then, a 5 mm × 5 mm craniotomy was performed on the right frontal skull and removed with a bone drill on the right skull bone 2 mm along the sagittal suture and 1 mm along coronal suture. A durotomy was performed, and 2 mm × 3 mm brain tissue was excised by sharp dissection (Yang et al., 2016). An electrocautery unit was used to stop bleeding, then the area was rinsed with normal saline. In the sham rats, the same surgical method was used; craniotomy was performed to remove the bone flap, but part of the right frontal lobe was not removed. Important parameters of interest were monitored during and after surgery. The rats were sacrificed at different time points according to experimental requirements.

### Drug Injection

The rats were randomly assigned into one of four groups, namely, Sham, SBI, SBI + vehicle, and SBI + BUM (15 mg/kg, 10% DMSO); rats in the SBI + BUM were treated via tail vein injection 15 min before SBI (Lu et al., 2006). The weight-matched SBI + vehicle group was injected with 10% equal volume of DMSO.

### Tissue Collection and Sectioning

The rats were anesthetized with sodium pentobarbital via intraperitoneal injection at 48 h post SBI (Figures 1A,B). To isolate proteins, the rats were perfused with 0.9% normal saline (200 mL, 4°C) through the heart, and cortical samples <3 mm from the contusion edge were collected on ice. Some tissue samples were flash frozen and stored at −80°C until analysis by WB and ELISA. To obtain brain sections, brains were harvested, fixed in 4% paraformaldehyde for >48 h at 4°C, followed by paraffin embedding. Paraffin brain sections were sectioned using a microslicer with a thickness of 5 μm each (Wu et al., 2020c). Tissue removal and selection were conducted by two blinded pathologists.

### WB Analysis

Western blotting (WB) analysis was performed as previously described (Tsai et al., 2020). The extracted brain tissue was homogenized in tissue protein extraction reagent with protease inhibitor cocktail (CWBIO, China) and incubated on ice for 20 min. Then, the homogenate was centrifuged at 12,000 *g* for 20 min at 4°C. The supernatant was collected, then the bicinchoninic acid (BCA) method and the Pierce^TM^ BCA protein detection kit (Thermo Fisher Scientific, United States) were employed to determine total protein concentration. Equal amounts of the extracted proteins were loaded and resolved by electrophoresis on a TGX Stain-Free FastCast Acrylamide Kit (Bio-Rad, United States), and then transferred onto a PVDF membrane (Millipore, United States). QuickBlock^TM^ Western (Beyotime, China) was employed to block the PVDF membrane at room temperature for 30 min and then sealed for 30 min at room temperature. The sections were then incubated with primary antibodies in a refrigerated shaker at 4°C overnight. The antibodies used were mouse anti-NKCC1 (Santa Cruz, CA, United States), rabbit anti-p-NKCC1 (Sigma, United States), rabbit anti-Albumin (Abcam, United Kingdom), rabbit anti-p-p65-NF-κB (Abcam), rabbit anti-p65-NF-κB (Abcam), rabbit anti-IL-1β (Abcam), rabbit anti-IL-6 (Abcam), and rabbit anti-TNF-α (Abcam), rabbit anti-caspase-3 (Abcam); Rabbit anti-GAPDH (Sigma) was used as internal loading control. After washing PBS thrice, the sections were incubated with secondary antibodies, which included anti-mouse IgG, HRP (Cell Signaling, United States), and anti-rabbit IgG-HRP (Invitrogen, United States) for 2 h at 4°C. The immunoblots were then stained with Immobilon^®^ Western Chemiluminescent HRP Substrate (Millipore, United States) and visualized using an imaging system (GE Healthcare Bio-Sciences AB, Sweden). The data were analyzed by ImageJ software (National Institutes of Health, MD, United States).

### Immunofluorescence Staining

Double IF staining was performed as earlier described (Zhao et al., 2016). A paraffin section oven was set at 70°C for 1 h. After soaking in xylene, anhydrous ethanol, 95%, 85%, and 70% ethanol, sodium citrate was used for repair. Then, the sections were washed thrice with phosphate-buffered physiological saline (PBS) after the membrane was permeabilized using an immunostaining permeable solution (Beyotime, China). The brain sections were blocked for at least 30 min in a blocking solution, then incubated with the indicated primary antibody at 4°C overnight. After three PBS washes, the sections were incubated with a secondary antibody at room temperature for 1 h. Finally, the tablets were sealed with DAPI anti-fluorescence quenching solution (YEASEN, China) and observed under a fluorescence microscope (OLYMPUS, Japan). Antibodies: rabbit anti-p-NKCC1 (Sigma, United States), rabbit anti-p-p65-NF-κB (Abcam), rabbit anti-NF-κB (Abcam), rabbit anti-IL-1β (Abcam), rabbit anti-IL-6 (Abcam), and rabbit anti-TNF-α (Abcam), mouse anti-GFAP (Invitrogen, United States), goat anti-Iba1 (Abcam), mouse anti-NeuN (Abcam), donkey anti-rabbit IgG antibody, Alexa Fluor 488 (Invitrogen), donkey anti-mouse IgG antibody, Alexa Fluor 555 (Invitrogen), donkey anti-goat IgG antibody, Alexa Fluor 555 (Invitrogen).

### Neurological Score

Neurological scoring was conducted as previously described (Feng et al., 2017) and consisted of the following seven components: (1) symmetry of limb movement; (2) forelimb-stretching exercises; (3) lateral turning; (4) climbing; (5) body movements; (6) responses to vibrissae touch; and (7) proprioception. Scores on each subtest ranged from 0 to 3, with a combined maximum score of 21. Higher scores were indicative of less neurological damage.

### ELISA

Interleukin-1β (IL-1β), IL-6, and TNF-α expression levels around the damaged rats brain areas were assessed by ELISA with a rat IL-1β, IL-6, TNF-α kit (BOSTER, China). This assay was conducted following the manufacturer’s instructions, and the collected data were expressed relative to a generated standard curve of IL-1β, IL-6, and TNF-α.

### TUNEL Staining

Apoptosis was determined via TUNEL staining following the manufacturer’s protocol (Beyotime, China). The paraffin-section dewaxing steps were the same as those described above. The samples were washed with distilled water for 2 min, followed by incubation for 30 min in a protease-K working solution. Later, the samples were washed thrice with PBS (5 min each wash). The brain sections were overlayed with TUNEL working solution and then incubated in a humidified box at 37°C for 1 h in the dark. The samples were then washed thrice with PBS (5 min each wash) and sealed with DAPI anti-fluorescence quenching solution (YEASEN, China).

### FJC Staining

Fluoro-Jade C staining was conducted following the manufacturer’s instructions (Biosensis, United States). The samples were incubated in a paraffin section oven at 70°C for 1 h. Then, the samples were successively soaked in xylene, 100% ethanol, and 70% ethanol. Subsequently, the samples were washed twice with double-distilled H_2_0 (1 min each time) followed by washing with distilled water for 2 min. Then, nine parts of distilled water were mixed with one part of solution B (potassium permanganate); the slides were then incubated for 10 min. Thereafter, slides were washed with distilled water for 2 min. Then, nine parts of distilled water were mixed with one part of solution C, and the samples were incubated in the dark for 10 min. After washing thrice with distilled water, the samples were dried in a 60°C oven for at least 5 min. Then, the samples were soaked in xylene for 5 min. After drying, the samples were sealed with neutral resin in liquid (YEASEN) and observed under a fluorescent microscope.

### Brain Edema

Brain edema was assessed, and brain moisture content was assessed using the wet-dry method. After separating the rat brain tissues, these were sectioned into symmetrical sides and their wet weights were immediately determined. Then, the brain samples were dried in a 100°C oven for 24 h, followed by dry weight determination. Brain water content (%) was estimated as follows: [(Wet weight - Dry weight) / (Wet weight)] × 100% (Wang et al., 2015).

### Statistical Analyses

All data are expressed as the mean ± standard deviation (SD). Statistical analysis was performed using GraphPad Prism 8.0 software. K–W One-way ANOVA for multiple comparisons as well as Student–Newman–Keuls *post hoc* tests were employed to determine significant differences among study groups. Differences with a *P* < 0.05 were considered statistically significant.

## Results

### Changes and Localization of NKCC1 and p-NKCC1 Protein Expression Levels in the SBI Brain

**FIGURE 2:**
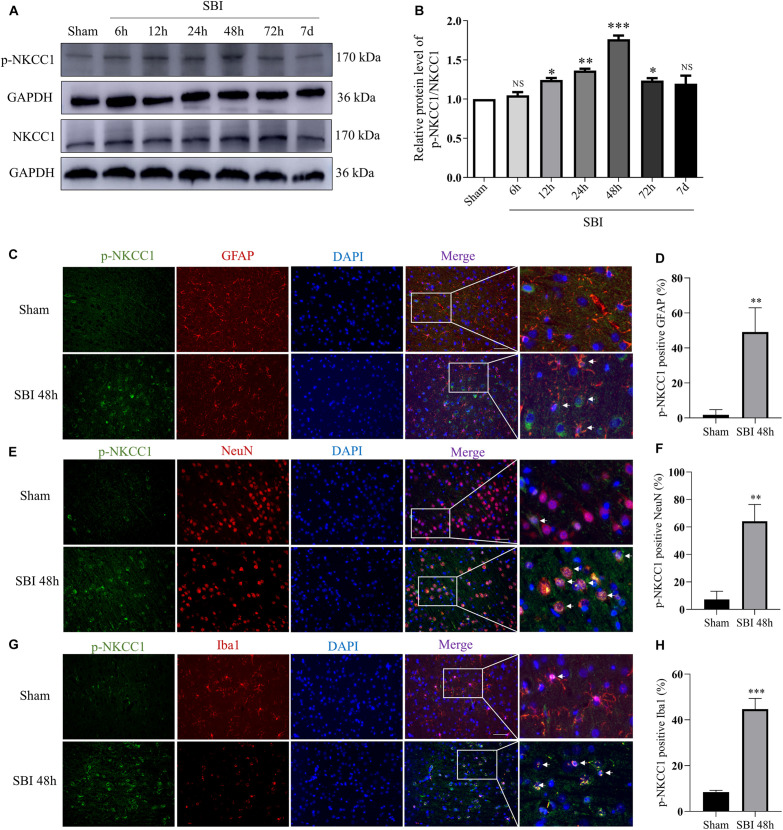
Expressions and localization of NKCC1, p-NKCC1 in lesions of the peripheral cortex after SBI. NKCC1/p-NKCC1 protein expression levels at 6 h, 12 h, 24 h, 48 h, 72 h, and 7 days in the SBI and sham groups were assessed by western blotting **(A,B)**. The relative densities of each protein were normalized to the sham group. ImageJ software was employed for quantification of protein expression levels, with the mean value of the sham group normalized to 1. Statistical analysis was conducted using a K–W one-way ANOVA, followed by the Student–Newman–Keuls *post hoc* test. In the sham group and 48 h after SBI, the expression of green-labeled p-NKCC1 and red-labeled GFAP **(C,D)**, NeuN **(E,F)**, Iba1 **(G,H)** were assessed by double IF. The nuclei were stained with DAPI (blue) fluorescence. Arrows show p-NKCC1 co-localization with astrocytes, microglial cells, and neurons. Scale bar = 50 μm. Statistical analyses were conducted using Student’s *t*-tests, *n* = 4 for each group. *P < 0.05, **P < 0.01, ***P < 0.001, ns *P* > 0.05, compared to the sham group.

Na^+^-K^+^-Cl^–^ cotransporter 1 (NKCC1) and p-NKCC1 expression levels were assessed at 6 h, 12 h, 24 h, 48 h, 72 h, and 7 days in the SBI and sham groups by WB and the changes of p-NKCC1 in cortical cells before and after SBI (Figure 2). p-NKCC1 expression began to rise 12 h after SBI and peaked at 48 h. After 48 h, protein levels and p-nkcc1 decreased. By 7 days, protein expressions levels returned back to normal, whereas that of NKCC1 did not change (Figures 2A,B). P-NKCC1 expression was evaluated by IF staining of GFAP, NeuN, and Iba1. The analysis showed that the number of p-NKCC1-positive astrocytes (Figures 2C,D), neurons (Figures 2E,F), and microglia (Figures 2G,H), were higher at 48 h in the SBI group relative to the sham group.

### Effects of BUM Intervention on NKCC1/NF-κB Signaling Pathway and Inflammatory Cytokines in Microglia After SBI

**FIGURE 3:**
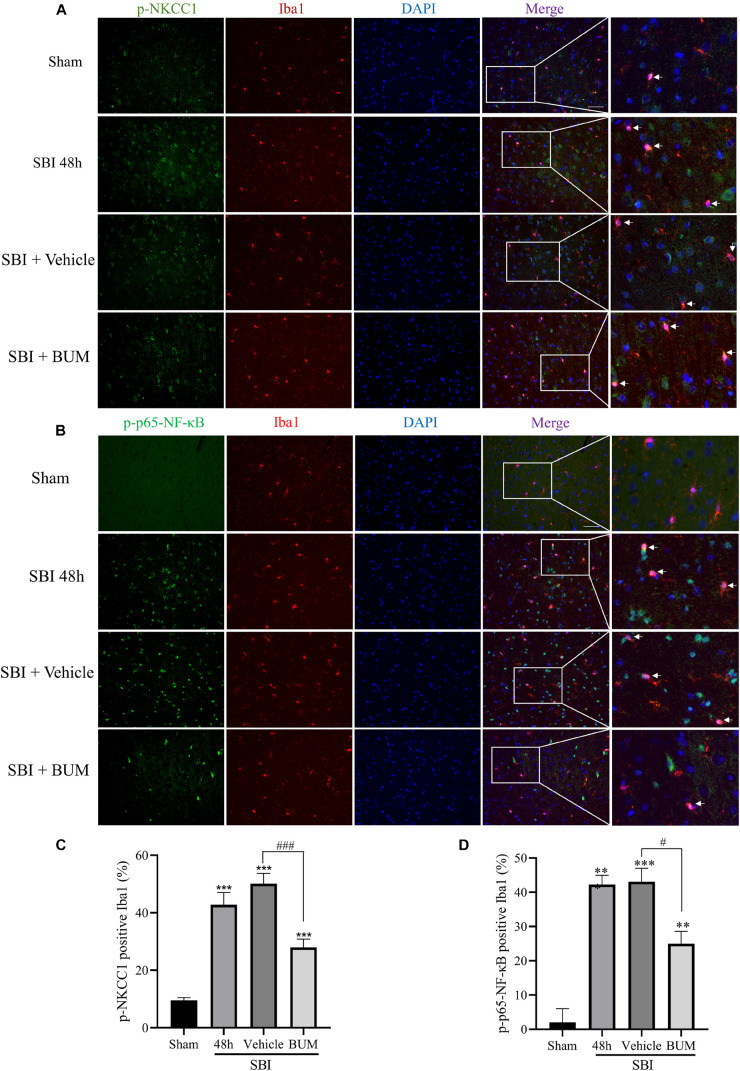
Double IF staining of p-NKCC1 and p-p65-NF-κB in the peripheral cortex of the damaged area. In the sham group, SBI group, SBI + vehicle group and SBI + BUM group, the expression of green-labeled p-NKCC1 **(A,B)**, p-p65-NF-κB **(C,D)** and red-labeled Iba1 were assessed by double IF. The nuclei were stained with DAPI (blue) fluorescence. Arrows show p-NKCC1 and p-p65-NF-κB co-localization with microglial cells. Scale bar = 50 μm. Statistical analysis was conducted using a K–W one-way ANOVA, followed by the Student–Newman–Keuls *post hoc* test. *n* = 4 for each group. **P < 0.01, ***P < 0.001, compared with sham group. ^#^*P* < 0.05, ^###^*P* < 0.001, compared with the SBI + Vehicle group.

**FIGURE 4:**
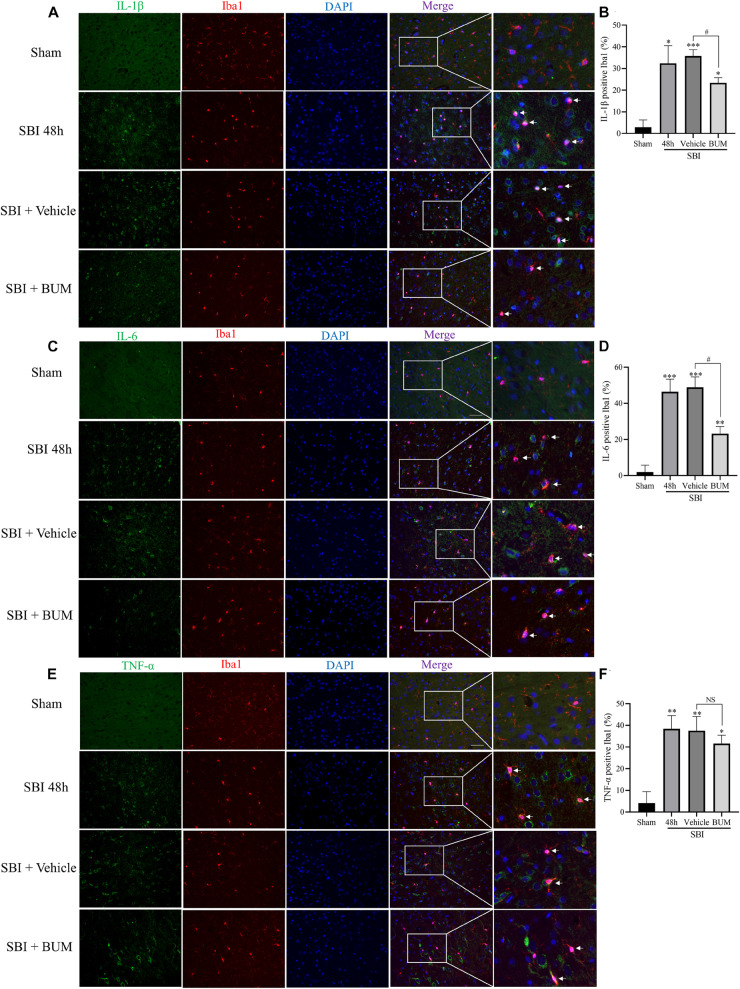
Double IF staining of IL-1β, IL-6, and TNF-α in the peripheral cortex of the damaged area. In the sham group, SBI group, SBI + vehicle group, and SBI + BUM group, the expression of green-labeled IL-1β **(A,B)**, IL-6 **(C,D)**, TNF-α **(E,F)**, and red-labeled Iba1 were assessed by double IF. The nuclei were stained with DAPI (blue) fluorescence. Arrows show IL-1β, IL-6, and TNF-α co-localization with microglial cells. Scale bar = 50 μm. Statistical analysis was conducted using a K–W one-way ANOVA, followed by the Student–Newman–Keuls *post hoc* test. *n* = 4 for each group. **P < 0.01, ***P < 0.001, compared with sham group. ^#^*P* < 0.05, compared with the SBI + Vehicle group.

Compared with sham group, the expressions of p-NKCC1 and p-p65-NF-κB in microglia of the SBI group, SBI + vehicle group, and SBI + BUM group were significantly increased. After BUM intervention, the expressions of p-NKCC1 and p-p65-NF-κB in microglia in the SBI + BUM group were decreased compared with the SBI + vehicle group (Figures 3A–D). Compared with sham group, the expression of TNF-α in the SBI group, SBI + vehicle group, and SBI + BUM group were significantly increased. After BUM intervention, TNF-α in microglia of the SBI + BUM group was not significantly changed compared with that of the SBI + vehicle group (Figures 4E,F). The variation trend of IL-1β (Figures 4A,B) and IL-6 (Figures 4C,D) in microglia was basically consistent with that of p-NKCC1/p-p65-NF-κB. These results confirmed that the increase of p-NKCC1 and p-p65-NF-κB in microglia of SBI stimulated the release of a large number of inflammatory cytokines from microglia, and BUM intervention could alleviate this process These results confirmed that a large number of inflammatory cytokines were released after SBI. BUM intervention can alleviate this process.

### BUM Intervention on the Expression of Cortical Related Proteins After SBI

**FIGURE 5:**
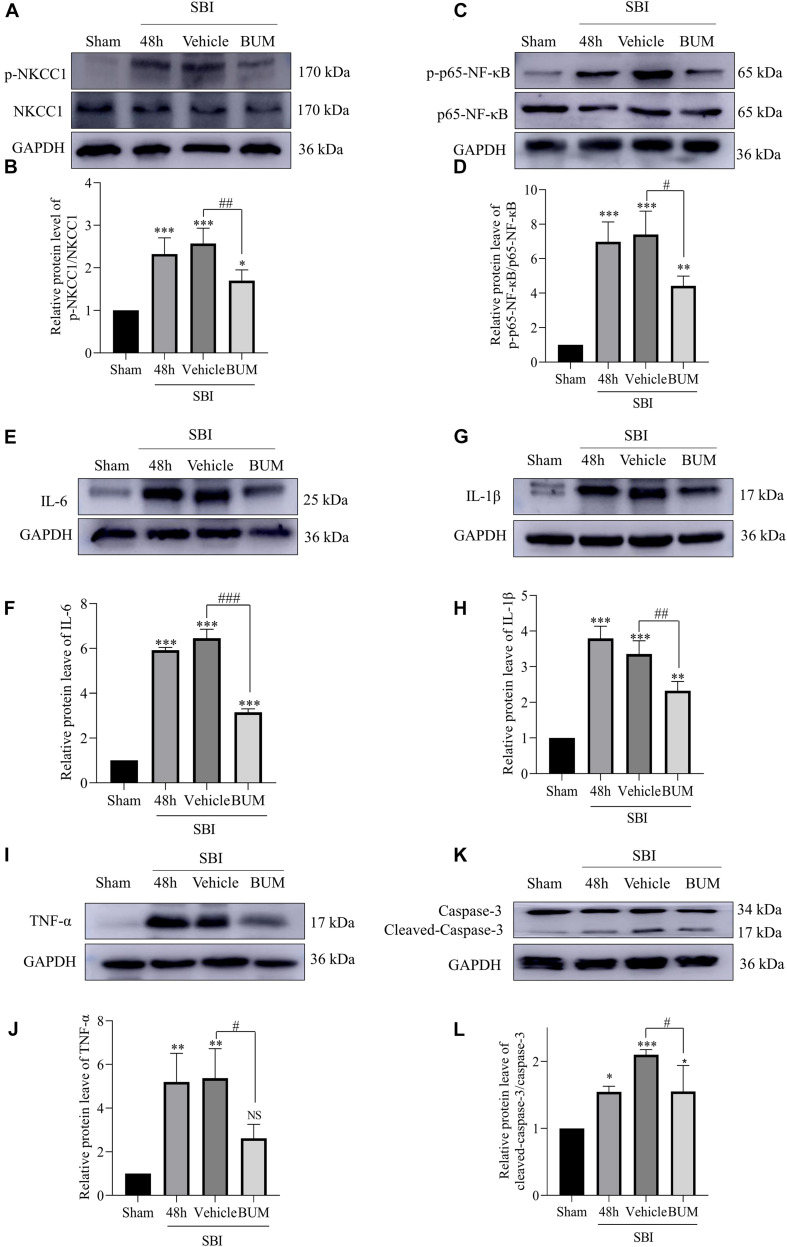
Expression levels of related proteins after the intervention of BUM 48 h after SBI. The protein-expression levels of p-NKCC1 **(A,B)**, p-p65-NF-κB **(C,D)**, IL-6 **(E,F)**, IL-1β **(G,H)**, TNF-α **(I,J)**, and caspase-3 **(K,L)** were assessed to determine the effect of BUM intervention on peripheral cortex injury 48 h post SBI. Statistical analyses were performed by a K–W one-way ANOVA followed by the Student–Newman–Keuls *post hoc* test. *n* = 4 for each group. **P < 0.01, ***P < 0.001 compared to the sham group. #*P* < 0.05, ##*P* < 0.01, ###*P* < 0.001 compared to the SBI + vehicle group.

Compared with the sham group, p-NKCC1, and p-p65-NF-κB expression markedly increased in the SBI group, SBI + vehicle group, and SBI + BUM group. After BUM intervention, WB showed that compared with the SBI + vehicle group, p-NKCC1, and p-p65-NF-κB expression in the SBI + BUM group decreased (Figures 5A–D). Compared with the sham group, TNF-α expression in the SBI group and SBI + vehicle group significantly increased. After intervention by BUM, WB showed significantly lower TNF-α expression in the SBI + BUM group compared with the SBI + vehicle group (Figures 5I,J). The trend for caspase-3, IL-1β, and IL-6 were roughly the same as that of p-p65-NF-κB (Figures 5E–H,K,L). These results confirmed that after SBI, inflammatory factors were released in large quantities, neuroinflammation was aggravated, and inflammation significantly improved after BUM intervention.

### ELISA, Brain Edema, and Neurological Behavioral Scores in SBI Rats After BUM Intervention

**FIGURE 6:**
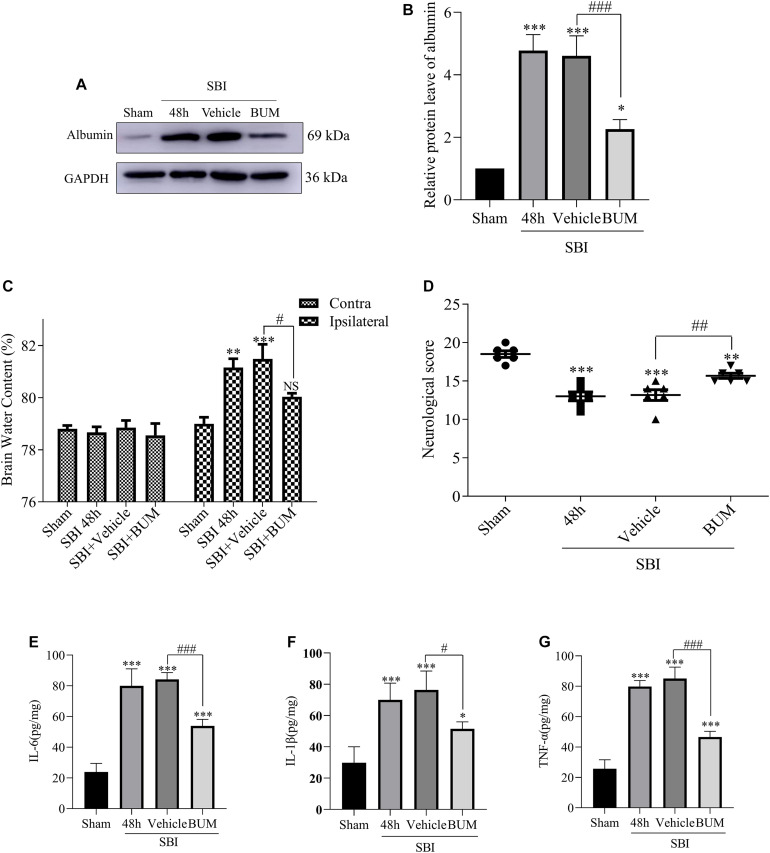
Effect of BUM intervention on ELISA, brain edema and neurological score 48 h after SBI surgery. The protein expression levels of albumin **(A,B)** were measured to evaluate the effect of BUM intervention on peripheral cortex injury 48 h after SBI. Brain water content of the bilateral hemispheres of the study groups was determined using the wet-dry method **(C)**. Neurological behavioral scores in the SBI rats after BUM intervention **(D)**. The ELISA expression levels of IL-6 **(E)**, IL-1β **(F)** and TNF-α **(G)** were assessed to determine the effect of BUM intervention on peripheral cortex injury 48 h after SBI. Statistical analyses were conducted using a K–W one-way ANOVA and the Student–Newman–Keuls *post hoc* test. *n* = 4 for each group. **P < 0.01, ***P < 0.001, compared with the sham group. #*P* < 0.05, ##*P* < 0.01, ###*P* < 0.001, compared with the SBI + Vehicle group.

Compared with the sham group, albumin expression in the SBI group and SBI + vehicle group and SBI + BUM group significantly increased. After BUM intervention, WB showed lower albumin expression in the BUM group compared with the SBI + vehicle group (Figures 6A,B). In addition, after SBI, cerebral edema in damaged hemispheres was significantly reduced by BUM intervention. However, there was no significant change in cerebral edema lateral to the SBI site of injury compared with the sham group (Figure 6C). Neurological scores of the SBI and SBI + vehicle groups were significantly lower than the sham group. Compared with the SBI + vehicle group, the neurobehavioral scores of the SBI + BUM group significantly improved. These results indicate that neurobehavioral scores significantly improved after SBI + BUM intervention (Figure 6D). ELISA was detected, compared with the sham group, IL-1β, IL-6, and TNF-α expression in the SBI and SBI + vehicle groups significantly increased. After BUM intervention, ELISA showed significantly lower IL-6 (Figure 6E), IL-1β (Figure 6F), TNF-α (Figure 6G) expression levels in the BUM group compared with the SBI + vehicle group.

### Effect of BUM Intervention on Neuronal Apoptosis and Degeneration Post SBI

**FIGURE 7:**
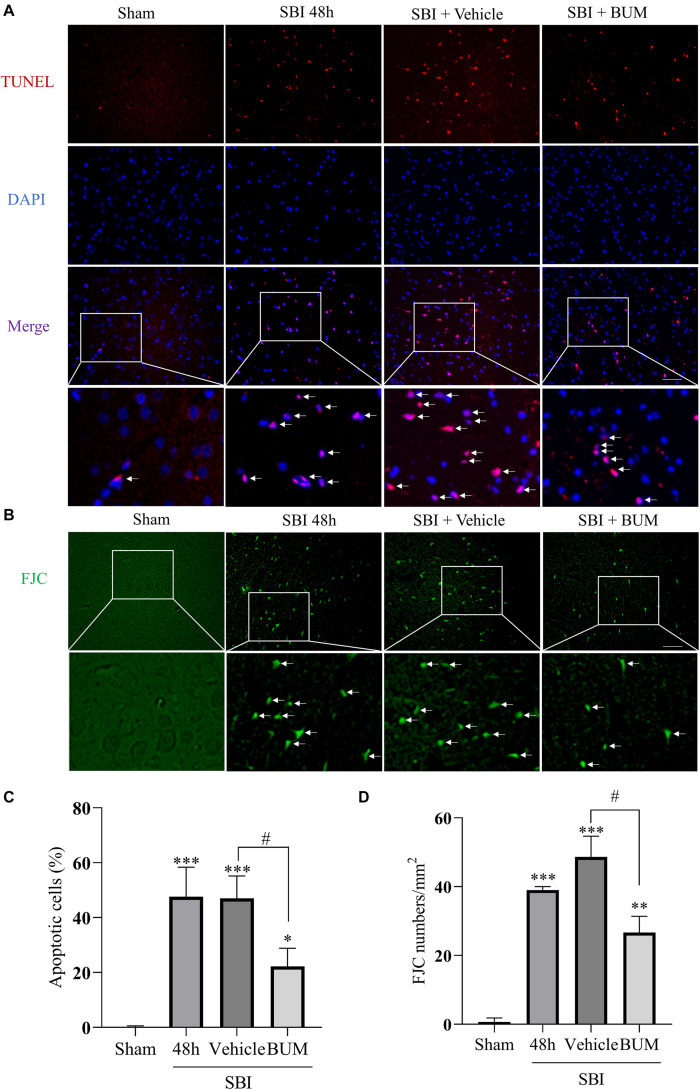
Effects of BUM intervention on peripheral cortical cell degeneration and apoptosis 48 h after SBI. Neuronal apoptosis was assessed by TUNEL staining **(A,C)**. TUNEL (red) labeled apoptosis and DAPI (blue) labeled nuclei. FJC (green) staining detected cell denaturation **(B,D)**. Scale bar = 50 μm. Statistical analysis was conducted using K–W one-way ANOVA, then with a Student–Newman–Keuls *post hoc* test. *n* = 4 for each group. **P < 0.01, ***P < 0.001, compared with the sham group. #*P* < 0.05, compared with the SBI + vehicle group.

The extent of apoptosis and neuronal degeneration in the SBI group and SBI + vehicle group and SBI + BUM group were marked higher than the sham group; however, no significant differences in these parameters were observed between the SBI and SBI + vehicle groups. Additionally, the extent of degeneration and apoptosis in the SBI + BUM group significantly decreased compared to the SBI + vehicle group (Figure 7).

## Discussion

In this study, we investigated the neuroprotective effect of inhibiting NKCC1 in the SBI rat model and explored its potential mechanism. Our study showed that NKCC1 expression did not show any significant changes after SBI, and p-NKCC1 protein levels began to rise 12 h after SBI and peaked at 48 h (Figure 2), subsequently decreased, and basically returned to normal levels on the 7th day. This suggested that NKCC1 plays a role via phosphorylation rather than inducing a total increase in the rat SBI model. This result is concordant to the findings of a previous study (Wu et al., 2020a). We found that BUM inhibition of NKCC1 can reduce the phosphorylation of NF-κB, reduce the release of inflammatory factors (IL-1β, IL-6, and TNF-α), reduce degeneration and apoptosis of nerve cells around the damaged area, improve nerve function, and reduce brain edema (Figures 4–7). This suggested that the brain edema and neuronal apoptosis after SBI were mediated by the activation of the NKCC1/NF-κB signaling pathway.

Microglia are considered to be the macrophages of the central nervous system, involved in chronic inflammation. The inflammation induced by the immune response is an important factor of neuron death after brain injury (Liu and Hong, 2003). In the early brain injury, the microglia can produce proinflammatory medium (IL-1β, IL-6, TNF-α), nerve toxicity compounds, reactive oxygen species, nitric oxide and other harmful substances (Chen et al., 2015; Wu et al., 2017a). At present, the mechanism by which microglia regulate the release of inflammatory cytokines after SBI is not fully understood. Increasing evidence shown that the upregulated expression levels of NKCC1 and inflammatory mediators including IL-1β, IL-6, and TNF-α were closely related to the development of secondary brain injury (Huang et al., 2014). Study have shown that inhibition of NKCC1 in microglia can reduce NF-κB and thus reduce inflammation after TBI (Zhang et al., 2017). Our study found that p-NKCC1 and p- NF-κB expression in microglia were significantly increased after SBI, leading to increased release of inflammatory factors such as IL-1β, IL-6, and TNF-α by microglia. After BUM intervention, p-NKCC1 in microglia cells was decreased, suggesting that BUM could inhibit the activity of NKCC1 in microglia cells. Moreover, p-p65-NF-κB was also reduced in microglia, which suggested that NF-κB was at least partially regulated by NKCC1 in SBI rat models (Figures 4, 5). NF-κB has been recognized as a central mediator of inflammatory processes and a key participant in innate and adaptive immune responses (DiDonato et al., 2012). By detected NF-κB signaling and downstream inflammatory cytokines, we found that reduction of p-p65-NF-κB inhibited the release of inflammatory cytokines in microglia. Inflammation damages the central nervous system, leading to an increase in apoptotic nerve cells (Takahashi et al., 2005). Under normal physiological conditions of brain tissue, microglia cells are dormant. After brain injury, microglia are activated and release inflammatory cytokines IL-1β, IL-6, and TNF-α(Huang et al., 2020b). This allows inflammatory substances to leak into the brain parenchyma, causing widespread neuroinflammation, leading to brain edema, and impaired nerve function (Haruwaka et al., 2019). Our study has shown that after SBI, microglia cells release a large number of inflammatory cytokines, causing neuroinflammation, leading to degeneration and increased apoptosis of nerve cells around the brain damaged area. Large amounts of nerve cell apoptosis can result in impaired nerve function. In addition, the apoptosis of nerve cells can destroy the BBB and aggravate the secondary brain edema after SBI.

Na^+^-K^+^-Cl^–^ cotransporter 1 (NKCC1) belongs to the cation-Cl^–^ Cotransporters and is an important determinant of ion homeostatics in brain. In the central nervous system, intracellular chloride concentrations are determined by NKCC1 and KCC2, and the two chloride ion channels have been identified as novel targets for the treatment of cerebral edema (Lee et al., 2003; Tian et al., 2015). By phosphorylating NKCC1, ions flow into the cell. Coordination across membrane ion and water flow of microglia and other nerve cells in the volume of the steady state is also necessary. As an ion regulatory protein, NKCC1 regulates of cell permeability changes (Matchett et al., 2006). In this study, we found that the expression of albumin and brain water content increased in the rat SBI model several hours post SBI. In fact, this may be due to the increase in p-NKCC1, which regulates the entry of substances such as Na^+^, K^+^, CI^–^, and water into cells, change cell volume, and destroy the cytoskeletal structure of cells, leading to cell swelling, increased BBB permeability, and albumin leakage, ultimately resulting in brain edema. NKCC1/NF-κB signaling affected secondary brain injury after SBI by regulating inflammation. Activation of this signaling pathway promoted microglia to release a large number of inflammatory mediators. At the same time, p-NKCC1 could open the channels on the cell membrane, increase the cell volume, and expand the space between the cell membranes. This promoted the transport of inflammatory cytokines, lead to swelling and apoptosis of cell, destructed of BBB, aggravated of brain edema. Our study showed that after inhibiting p-NKCC1, compared with the injured group, albumin exudation, cerebral water content, and inflammatory factors of the injured side of rats with cerebral edema as well as apoptosis significantly decreased (Figure 7). These results suggest that inhibiting p-NKCC1 can reduce neuroinflammation in SBI rats, protect BBB, and improve the apoptosis of nerve cells, thereby reducing SBI-induced brain edema and imparting a protective role in the brain.

**FIGURE 8:**
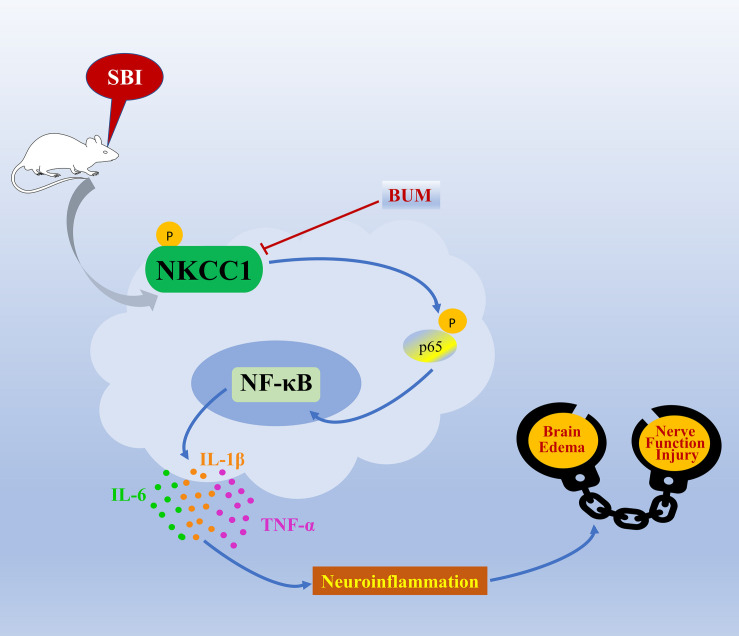
A model illustrating the possible mechanisms by which NKCC1 influences SBI by phosphorylation of NF-κB. After SBI, microglia cells are activated, p-NKCC1 expression rapidly increases, and NF-κB in the nucleus is phosphorylated. The increase in p-NF-κB expression triggers the release of IL-6, IL-1β, and TNF-α inflammatory factors, which leads to neuroinflammation, damages the BBB, ultimately leading to brain edema and nerve function injury.

The mechanism of SBI causing secondary brain injury is highly complex. According to our research, p-NKCC1 in microglia increased greatly after SBI, and the phosphorylation of downstream p-p65-NF-κB signal increased accordingly. Microglia cells are activated and release a large number of inflammatory factors, leading to neuroinflammation. Meanwhile, as an ion-regulating protein, NKCC1 can increase the volume and permeability and water content of cells, promote the diffusion of inflammatory factors, aggravate inflammation, lead to degeneration and apoptosis of nerve cells, and cause brain edema and neurological dysfunction. Inhibition of NKCC1/NF-κB signaling pathway can alleviate secondary brain injury caused by SBI (Figure 8).

However, this study is limited by the small sample size and use of only male rats. Thus, we were unable to assess gender differences in p-NKCC1 expression after SBI. Furthermore, to further verify our results, we did not study whether upregulation of p-NKCC1 aggravates SBI-induced secondary brain injury. We will continue investigating the above deficiencies in future studies.

## Conclusion

Here, we show that after SBI in rats, NKCC1 is activated, phosphorylates downstream p-p65-NF-κB, and promotes microglia to secrete IL-1β, IL-6, TNF-α, which aggravates secondary brain injury. Inhibiting the activity of NKCC1 induces protective effects on the brain. These results indicate that NKCC1 may be potentially used as a prevention and control target for SBI.

## Data Availability Statement

The original contributions presented in the study are included in the article/supplementary material, further inquiries can be directed to the corresponding authors.

## Ethics Statement

The animal study was reviewed and approved by the Institute of Animal Care Committee of Zhangjiagang Traditional Chinese Medicine Hospital.

## Author Contributions

GC and BD conceived and designed the study. YG and MW conducted the experiment and wrote the manuscript. JS helped in capturing and processing micrographs. JT and JL assisted in the literature search and conducted the statistical analysis. JX reviewed and revised the manuscript. All authors have read and approved the submission of the manuscript.

## Conflict of Interest

The authors declare that the research was conducted in the absence of any commercial or financial relationships that could be construed as a potential conflict of interest.
